# Self-reported and parent proxy reported functional impairment among pediatric cancer survivors and controls

**DOI:** 10.1186/s12955-020-01387-z

**Published:** 2020-05-18

**Authors:** Sarah J. Erickson, Sarah Hile, Nicole Kubinec, Robert D. Annett

**Affiliations:** 1 Department of Psychology, Logan Hall, University of New Mexico, MSC03 2220, Albuquerque, NM 87131 USA; 2 Private practice, Albuquerque, NM USA; 3 Department of Pediatrics, University of Mississippi Medical Center, Jackson, MS USA

**Keywords:** Childhood Cancer survivors, Executive function, Functional impairment, Neurocognitive deficits, Pediatric oncology, Self-report, parent proxy report, Brief impairment scale

## Abstract

**Background:**

A unique and limiting component in the research on functional impairment among children has been the exclusive use of parent proxy reports about child functioning; and there is limited information regarding the impact of pediatric cancer treatment on children’s day-to-day functioning and how this is related to neurocognitive functioning. The objective of the current study was to examine a novel measure of self-reported functional impairment, and explore the relationship between self-reported and parent-reported child functional impairment in pediatric cancer survivors compared to controls.

**Methods:**

A cross-sectional cohort of survivors (*n* = 26) and controls (*n* = 53) were recruited. Survivors were off treatment an average of 6.35 years (*SD* = 5.38; range 1–15 years) and demonstrated an average “medium” Central Nervous System treatment intensity score. Participants completed measures of functional impairment (FI), intellectual assessment (RIST) and executive functions (NIH Examiner), while parents reported on children’s functional impairment.

**Results:**

Survivors were similar to controls in functional impairment. Regardless of group membership, self-reported FI was higher than parent-reported FI, although they were correlated and parent report of FI significantly predicted self-reported FI. Across groups, increased impairment was associated with four of seven Examiner scores.

**Conclusions:**

Research regarding self-reported functional impairment of cancer survivors and its association with parent-reported functional impairment and neurocognitive deficits has been limited. Our results suggest that self-reported FI appears to be a reasonable and viable outcome measure that corresponds with and adds incremental validity to parent reported FI. While low treatment intensity may confer relative sparing of functional impairment among survivors, children report higher FI levels than parents, suggesting that FI can be of clinical utility. In conclusion, pediatric cancer survivors should be screened for self-reported functional difficulties.

The importance of day-to-day functioning in the context of children’s physical and psychological health has emerged as a research focus [1]. However, limited information exists regarding the impact of pediatric cancer treatment on children’s day-to-day functioning. Functional impairment (FI), defined as limitations in managing life activities, has emerged from research involving children with attention deficits [2–4]. FI provides a benchmark beyond isolated deficits observed in neuropsychological or psychosocial domains and includes domains such as interpersonal functioning, school functioning, and self-care/self-fulfillment activities [5]. Specifically, FI shifts the focus from the cause to the impact of a disease on day-to-day functioning [6] and includes the ways in which a constellation, rather than isolated psychological symptoms, interferes with and reduces performance within important child domains [7]. In this regard, the importance of FI lies in its ability to capture activities that are salient to a child’s life. Three major realms in which daily functioning may be disrupted include: interpersonal relations (e.g., trouble making friends), school/work functioning (e.g., trouble getting schoolwork completed), and self-care/self-fulfillment (e.g., feelings about appearance) [5]. Previous research has indicated that FI represents an independent construct and should be distinguished from specific psychological symptoms [7].

Although FI has previously been linked to children with traumatic brain injury, attention deficits, and other mental health problems [2, 8–11], it has received limited attention in pediatric cancer survivors [12–14]. Survivors have been observed to demonstrate functional deficits, such as needing help with personal care and routine daily rituals, difficulty keeping and holding a job, have demonstrated difficulties with scholastic achievement, and are significantly less likely to complete high school [13]. Long-term findings from the Childhood Cancer Survivor Study (CSSS) have indicated higher risks for later employment difficulties and unemployed [14].

A potentially limiting component in FI research has been the exclusive use of parent proxy reports about child functioning. While parent reports of child functioning are contextually important, many children are capable of providing their own perspective on functioning, which has been evident in quality of life research where the child’s perspective has been assessed [15–17]. Research investigating child-parent concordance of *psychological* symptoms has identified discrepancies in symptom levels, clustering of symptoms, and caseness levels; and ways in which reporters provide unique perspectives [18]. In contrast, child perspectives have not been elicited in extant measures of functional impairment. Eliciting the perspective of the child can add important information not captured by parent report, thereby benefitting both researchers and clinicians in appreciating the extent to which the child is aware of and can report their management of activities which are often a focus of psychological intervention. Researchers in this area have identified benefits from assessing FI from both child and parent perspectives [1].

Linkage between child day-to-day functioning and neurocognitive function informs the broad landscape for psychological interventions that can impact overall quality of life. Child neurocognitive function importantly includes executive skills, which include the capabilities for responding adaptively to novel situations and forming the basis of many cognitive, emotional, and social skills [19]. Executive functioning has been implicated in broad functional outcomes such as school readiness [20], academic achievement [21], theory of mind, and social competence [22], all of which are relevant to successful post-treatment adaptation in pediatric cancer survivors. Campbell and colleagues [23] examined the impact of executive functioning domains on coping strategies and behavioral outcomes among survivors of Acute Lymphoblastic Leukemia (ALL), finding that performance on executive functioning tasks was related to coping strategies, as well as emotional and behavioral problems. Impaired task efficiency, emotion regulation, organization, and memory have been associated with lower socioeconomic achievement [24] and impaired attention has been related to poor math and reading achievement [25] and poor health related quality of life [26]. Together, these findings provide compelling evidence that impairments in executive functioning may impact functional domains including academic achievement, social and emotional competence, and socioeconomic achievement; however, additional research is needed to further elucidate this relationship.

The current study developed and tested a child self-report of FI (Brief Impairment Scale-Child; BIS-C), which was based upon an existing proxy report (Brief Impairment Scale; BIS). This project sought to achieve 6 goals: 1) examine the properties of a child self-report measure of FI; 2) compare self-reported FI scores between cancer survivors and controls; 3) compare self-reported and parent proxy reported FI scores; 4) determine the child age effect on self-reported FI scores; 5) examine the relationship between self-reported FI and neurocognitive functioning; and 6) explore predictors of self-reported FI. We hypothesized that BIS-C scores would distinguish cancer survivors from controls; BIS-C scores would correspond with parent-reported BIS scores and be related to child age; and that BIS-C scores would be associated with child neurocognitive functioning.

## Methods

### Participants

Pediatric cancer survivors were recruited from the University of New Mexico’s Pediatric Oncology Late Effects Clinic. Inclusion criteria were: 1) cancer diagnosis, 2) age 5–18, 3) at least one-year post-treatment, and 4) ability to follow instructions. Controls aged 5–18 years were recruited via multiple sources (online ads placed on Craigslist, flyers placed in the community, as well as word of mouth). Children were ineligible if they had impaired cognitive ability (IQ < 70), a chronic illness that could be expected to increase FI (e.g., seizure disorder or sensory impairment), or limited English proficiency. A recruitment pool of 108 survivors was approached to participate and 26 agreed (recruitment rate of 24%). We recruited and evaluated for case-control analyses but did not have adequate matching between the groups (only 17 survivors matched in age with controls); therefore, all 53 controls were included in subsequent analyses. All control participants who responded to study advertisements in the community met eligibility criteria based on a telephone screening.

### Procedure

Study procedures were approved by the University of New Mexico Institutional Review Board. Following assent/consent, children and parents were separated, with children completing measures (neurocognitive and BIS-C) while parents completed questionnaires (BSMSS, BIS, and a demographic questionnaire (child age, child sex, child ethnicity, receiving Supplemental Security Income; and for the cancer survivor group: treatment intensity, time since end of treatment)), using previously validated questions [27]. A neurocognitive and executive function exam was administered to the child by a trained psychometrist. After completion of the neurocognitive exam, the child completed a questionnaire assessing functional impairment. Questions were read aloud to the child who selected answer choices from a booklet. This process required approximately 60 min of the child’s time. Parents and children each received a gift card.

### Measures

#### Barratt simplified measure of social status (Hollingshead AB: Four factor index of social status, unpublished)

Socioeconomic status (SES) was measured using the Barratt Simplified Measure of Social Status (BSMSS) [28], with higher values being indicative of higher SES. The BSMSS is based on Hollingshead’ measure (Hollingshead AB: Four factor index of social status, unpublished; Hollingshead AA: Two factor index of social position, unpublished), a simple measure of social status based on marital status, occupation, educational attainment, and occupational prestige. The BSMSS is a proxy for SES based on two factors: parent(s)’ total education and occupation.

#### Functional impairment

Functional Impairment was assessed with the Brief Impairment Scale (BIS) [5]. The BIS is a 23-item parent-completed assessment that provides a global measure of impairment along three domains of functioning: interpersonal relations, school/work functioning, and self-care/self-fulfillment. The measure assesses the degree to which the child struggles with various activities. Responses are on a 4-point Likert scale ranging from 0 (“no problem”) to 3 (“serious problem”). Content structure of the BIS further allows for questions to be refused, not applicable, and “don’t know.” The assessment is prefaced by the statement “In general, how much of a problem do you think your child has with.” It then includes item statements such as: “Getting involved in activities together with the rest of the family,” “Making friends,” and “Getting schoolwork done on time.” Based upon large multi-national community and clinical samples, two cutoff scores (i.e., scores of 11.5 or greater; and scores of 14 or greater) were developed using receiver operating characteristics to identify clinically significant impairment or “caseness” [5]. Convergent validity, including item representativeness, was demonstrated by significant correlations (*r* = − 0.53, 0.52, and − 0.52; *p* < .001) between the BIS and an established measure of the same construct, the CGAS [29]. Concurrent validity was demonstrated by significant mean BIS score differences between clinical and non-clinical samples. Test-retest reliability is moderate, with total scale ICC = .070 and subscale agreement fair to moderate (interpersonal ICC = .56, school ICC = .54, self ICC = .76). The BIS has internal consistency with Cronbach’s alpha ranging from .81 to .88, as well as fair to substantial test-retest reliability.

We sought to further enhance the measurement of functional impairment by adapting the BIS so that it could be completed by children (Brief Impairment Scale-Child Version; BIS-C). Thus, we sought to determine the extent to which children were able to report on their own functional status. The BIS-C was based on the same 4-point Likert scale as the original version with options for refusal, not applicable, and “don’t know.” Similar to the original, items were introduced with the statement, “In general, how much of a problem do you think you have with.”

For this preliminary study, BIS-C item content was examined for face and content validity, as well as reading level, by the research team. Using team consensus methods, item content was slightly modified, including simplified language and examples for children. Example items include: “Getting involved in activities together with the rest of the family,” “Making friends,” and “Getting schoolwork done on time.” Reading level was assessed with the Flesch Reading Ease Score and adjusted to achieve a 5th grade reading level used in assent/consent documents.

#### General intellectual function

General intellectual ability was assessed using the Reynolds Intellectual Screening Test (RIST) [30]. This is comprised of two subtests and was administered to all children. The RIST was standardized on 2438 individuals in 41 states and is representative of the 2001 US Census. Reliability coefficients range from 0.84 to 0.96. Test-retest reliability ranged from 0.79 to 0.86. Correlations with the Wechsler Intelligence Scale for Children, Third Edition (WISC-III) Full Scale IQ were 0.76. The RIST can be completed by children in less than 20 min.

#### Executive function

Executive function was assessed using the NIH Examiner [31]. The Examiner assesses multiple domains of executive functioning including working memory, inhibition, set shifting, fluency, planning, insight, and social cognition/behavior. The Examiner also provides an executive function composite score as well factor scores across three sub domains: working memory, fluency, and cognitive control. The Examiner has demonstrated good psychometric properties. All tasks had appropriate internal consistency with alpha ranging from .64 to .98. Test-retest reliability across the executive function composite and factor scores ranged from 0.76 to 0.94. Convergent validity was demonstrated by significant correlations (r = − 0.21, *p* < .001) between the NIH examiner composite score and a measure of parent report of real-world executive function (BRIEF). The NIH Examiner was used to develop scores for Fluency (Verbal Fluency), Planning (Unstructured Task, Unstructured Task Weighted Composite), Cognitive Control (Continuous Performance Task (CPT) Targets Correct, CPT Target Errors), Working Memory (NBack), Set Shifting, and an Executive Composite score.

### Statistical analyses

All analyses were conducted using SPSS 25 [32]. Initial analysis of the BIS and BIS-C included examination of individual item scores. One item (Suspended from school in the past 12 months) had no variance so it was deleted in both BIS and BIS-C analyses. Responses that were coded as refusal, not applicable, and “don’t know” were coded as missing and subsequent interpolation for missing values resulted in *N* = 79. Alpha for the BIS was good (.81; 22 items) and for the BIS-C was acceptable (.69; 22 items). A 3-domain structure was proposed by Bird et al. [5] for the BIS, identifying three specific areas of impairment (e.g., interpersonal functioning, school functioning, and self-care/self-fulfillment). The rationally derived domain structure was maintained in the BIS-C analyses.

Characteristics of the BIS and BIS-C were examined with correlation and t-tests. For age analyses, child age was rationally divided into groups (5–7; 8–10; 11–14; 15–18 years) of roughly equal numbers of children. Assuming a small effect size (0.25), correlation between measures (0.50), and desired statistical power of 0.95, a sample size of 79 was sufficient for analyses.

Hierarchical linear regressions were conducted to determine the impact of predictors of BIS-C scores. We sought to test two models of predicting BIS-C scores. In the first model, age and SES were entered first, with parent-completed BIS total scores entered second. Age was included because although we found no significant (parent proxy) BIS total difference by age, the older the child, the greater the *tendency* towards greater BIS (FI) [33]. SES was included because life stressors, closely associated with SES, represent a significant predictor of functional impairment in children [34]. In the second regression model, all Examiner subscales were initially entered as a block and RIST IQ was entered second.

## Results

### Preliminary analysis

Descriptive statistics for 53 controls and 26 survivors are presented in Table 1. Cancer survivors were significantly older, with no other demographic differences evident. Survivors had ended treatment an average of 6.35 years (SD = 5.38; range 1–15 years) prior to participation. Among the survivor group, 62% of children had CNS-related cancers, while 38% had non-CNS cancers. Survivors qualified for a median CNS treatment intensity of 2.0 (Mean = 1.42; range 0–4) [27]. In fact, 17 of 26 survivors had an intensity score of 1–2 and 7 had no CNS treatment. BIS-C scores were not different for the survivor and control groups (Table 1), so the groups were combined for subsequent analyses. There was no effect of child sex on BIS-C total or subscales.

**Table 1:** Sociodemographic variables, illness severity, Brief Impairment Scale-Child (BIS-C), Brief Impairment Scale-Parent (BIS), Reynolds Intellectual Screening Test (RIST), and NIH Examiner

	Controls (*n* = 53) Mean (SD)	Cancer Survivors (*n* = 26) Mean (SD)	*p*-value
Child age (Years)	9.94 (3.64)	12.35 (3.99)	.009**
Child sex (Male)	53%	50%	.813
SES (Barrett score)^a^	37.19 (10.61)	33.47 (10.83)	.150
Child Ethnicity ^b^			.155
Hispanic	45%	46%	
White	23%	27%	
Receiving SSI ^c^	6%	8%	.727
Treatment Intensity ^d^	N/A	1.42 (0.99)	
Time since end of treatment (years)	N/A	6.35 (3.62)	
RIST^f^			
Total Index IQ	98.68 (13.58)	99.27 (12.88)	.854
Guess What	45.53 (11.85)	47.46 (10.00)	.476
Odd-Item-Out	51.35 (10.36)	50.62 (9.27)	.709
BIS-Child			
School	3.97 (2.85)	3.19 (2.98)	.264
Interpersonal	5.47 (3.75)	4.29 (3.84)	.199
Self	4.94 (2.75)	4.81 (2.40)	.831
Total	14.38 (6.18)	12.29 (7.16)	.184
BIS-Parent ^**e**^			
School	2.63 (2.70)	2.69 (3.03)	.822
Interpersonal	2.45 (2.18)	2.23 (2.27)	.985
Self	3.08 (2.61)	2.92 (2.95)	.690
Total	8.16 (6.13)	7.85 (6.28)	.979
NIH Examiner ^g^			
Verbal Fluency Total	21.48 (9.08)	26.04 (8.85)	.038*
Unstructured Task Total	15.40 (7.24)	19.08 (8.33)	.047*
Unstructured Task Weighted Composite ^h^	−6.53 (1.46)	−6.48 (2.08)	.905
CPT Targets Correct	72.69 (18.60)	77.65 (6.01)	.190
CPT Target Errors	1.83 (2.88)	1.96 (5.52)	.888
N-Back Score	1.61 (0.68)	1.78 (0.77)	.358
Set Shifting Score	6.68 (1.15)	7.08 (1.20)	.170
Executive Composite	−0.42 (5.33)	1.70 (6.43)	.134

^a^Barratt Simplified Measure of Social Status; Raw Score Range: 8–66

^b^Ethnicity: Hispanic and Non-Hispanic

^c^SSI: Supplemental Security Income; Percent yes

^d^Treatment Intensity (range 0–3)

^e^BIS: Brief Impairment Scale raw (0–69)

^f^Reynolds Intellectual Screening Test. Composite IQ is standard score; Guess What and Odd-Item-Out scores are T-scores

^g^NIH Examiner raw scores

^h^Weighted composite = UTpct*log10(UTTotal+ 1; UTpct = percentage of completed puzzles that were considered high value items; UTTotal = total number of points earned)

*p < .05, ** *p* < .01, ***p < .001

### Child report of FI compared with parent report of FI

BIS-C scores were significantly higher than BIS scores on total, interpersonal relations, school, and self-care/self-fulfillment subscales (t = 6.63, *p* = .000; t = 5.95 p = .000; t = 2.89, *p* = .005; and t = 4.71, p = .000, respectively) (Table 2). The BIS-C significantly correlated with BIS (r = .298, *p* = .008), and regarding correlations between like-subscales, the BIS-C school correlated with BIS school (r = .436, p = .000). Both BIS-C and BIS subscale scores were highly intercorrelated (Table 3).

**Table 2:** Brief Impairment Scale-Parent (BIS) and Brief Impairment Scale-Child (BIS-C) scores

	Mean (SD), *N* = 79	
	BIS	BIS-C
Total***	8.06 (6.14)	13.55 (6.58)
Interpersonal***	2.38 (2.20)	4.94 (3.74)
School**	2.65 (2.79)	3.76 (2.89)
Self-Care***	3.03 (2.71)	4.85 (2.61)
Caseness GE 11.5	18	47
Caseness GE 14*	11	37

The BIS is a parent-report measure of impairment in children that assesses areas of interpersonal functioning, school functioning, and self-care or fulfillment

The BIS-C is a self-report measure of impairment in children that assesses areas of interpersonal functioning, school functioning, and self-care or fulfillment

* p < .05; ** *p* < .01; *** *p* < .001

Caseness defined by BIS and BIS-C scores greater than or equal to 11.5 or 14

**Table 3:** Correlation matrix of child and parent Brief Impairment Scale (BIS) scores (*n* = 79)

Correlation coefficient	BIS-C Total	BIS-C Interpersonal	BIS-C School	BIS-C Self-Care
BIS Total	0.307**	0.175	0.332**	0.154
BIS Interpersonal	0.299**	0.197	0.338**	0.097
BIS School	0.357**	0.216	0.436***	0.108
BIS Self-Care	0.084	0.014	0.030	0.158

The BIS-C is a self-report measure of impairment in children that assesses areas of interpersonal functioning, school functioning, and self-care or fulfillment

The BIS is a parent-report measure of impairment in children that assesses areas of interpersonal functioning, school functioning, and self-care or fulfillment

*p < 0.05

**p < 0.01

***p < 0.001

BIS-C caseness was not related to sex or IQ (chi-square). When the BIS caseness cutoff was set at GE11.5, there was agreement for 28 dyads regarding non-casenesss and 14 dyads regarding caseness. Specifically, caseness was achieved for 47 children based on BIS-C; and 18 based on BIS, and this caseness discrepancy was at a trend-level, but non-significant (Chi square = 3.28, *p* = .07). When the BIS caseness cutoff was set at GE14, there was agreement between child and parent reports of FI for 40 dyads re: non-caseness and 9 dyads re: caseness. Specifically, caseness was achieved for 37 children based on BIS-C; and 11 based on BIS, and this caseness discrepancy was significant (Chi square = 6.28, *p* = 012) (Table 2).

### Age and survivor versus control group effects on BIS-C

**Fig. 1:**
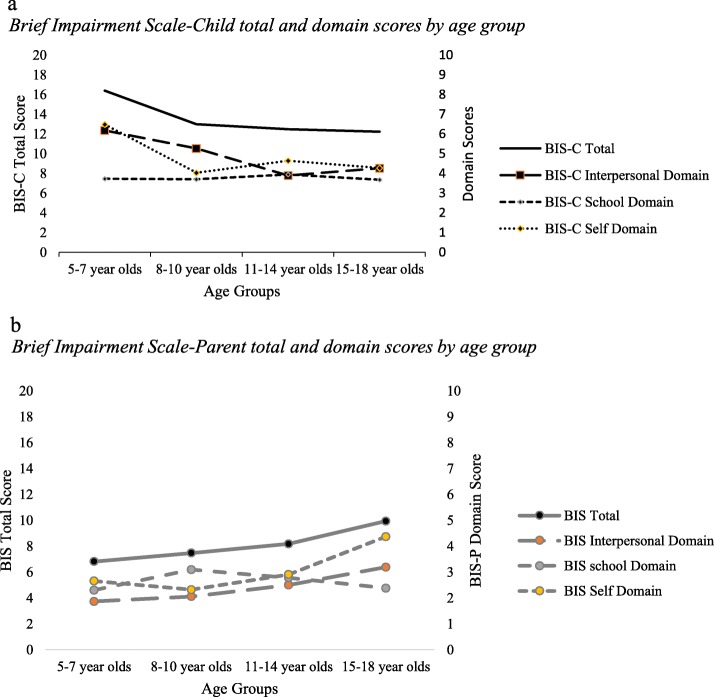
**a** Brief Impairment Scale-Child total and domain scores by age group. **b** Brief Impairment Scale-Parent total and domain scores by age group

To test our hypothesis regarding an age effect, four age groups were created for purposes of analysis (5–7; 8–10; 11–14; 15–18 years) (Table 4). No age group effect was observed on the BIS-C total score (F = 1.679; *p* = .095), but there were main effects for the BIS-C interpersonal relations and self-care/self-fulfillment domain scales (F = 4.155, *p* = .045; F = 4.725, *p* = .033, respectively) such that as age increased, children reported less impairment (Fig. 1a). Significant BIS-C subscale group differences were observed between 5–7 years and both 8–10 years and 15–18 years on the self-care/self-fulfillment subscale, with 5–7 years reporting greater self-care/self-fulfillment FI (*p* = .01, *p* = .032, respectively). These findings are contrary to our previous trend-level findings that as child age increases, *parents* report greater amounts of functional impairment [33] (Fig. 1b). There were also no group, group x age, or sex effects on BIS-C total and three subscales.

**Table 4:** Brief Impairment Scale-Child (BIS-C) scores and caseness percent by age group

	Mean (SD)				Caseness	
	BIS-C Total	BIS-C Interpersonal	BIS-C School	BIS-C Self-Care	GE 11.5	GE 14
5–7 years (*n* = 20)	16.40 (6.34)	6.18 (3.38)	3.73 (2.96)	6.49 (3.09)	80.0%	60.0%
8–10 years (*n* = 22)	12.99 (6.44)	5.26 (4.01)	3.70 (2.75)	4.03 (1.86)	54.5%	36.4%
11–14 years (*n* = 18)	12.47 (6.79)	3.89 (4.10)	3.94 (2.94)	4.64 (2.66)	50.0%	44.4%
15–18 years (*n* = 19)	12.22 (6.40)	4.26 (3.21)	3.68 (3.16)	4.28 (2.13)	52.6%	47.4%
Total (n = 79)	13.55 (6.58)	4.94 (3.74)	3.76 (2.89)	4.85 (2.61)	59.5%	46.8%

The BIS-C is a self-report measure of impairment in children that assesses areas of interpersonal functioning, school functioning, and self-care or fulfillment

Caseness defined by BIS-C scores greater than or equal to 11.5 or 14

### Relationship between FI and IQ/examiner

In addressing our hypothesis regarding the relationship between BIS-C and Examiner/IQ, correlations demonstrated that BIS-C correlated with Verbal Fluency (r = −.231, *p* = .026), CPT Target Errors (r = .270, *p* = .011), N-Back (r = −.238, *p* = .023), and Executive Composite (r = −.214, *p* = .037) in the predicted directions. BIS-C subscales and Examiner/IQ correlations revealed that the BIS-C interpersonal relations subscale correlated with CPT target errors (r = .297, *p* = .013); and the BIS-C self-care/self-fulfillment subscale correlated with RIST scores (r = −.253, *p* = .034). However, because age was associated with BIS-C and with Examiner scores, when age was controlled, all correlations lost significance.

### Predicting BIS-C scores

Table 5 presents the results from hierarchical linear regression modeling of predictors of BIS-C scores. The first model indicated that step 1 (SES and child age) was significant at a trend level (adjusted R^2^ = .049, F = 2.997, *p* = .056) and step 2 (BIS) was significant (adjusted R^2^ = .146, F = 5.449, *p* = .002). Child age and BIS were unique and significant predictors (Beta = −.269, *p* = .014; Beta = .339, *p* = .003, respectively) (Table 5). The second regression model (Examiner and RIST IQ) revealed that step 1 (Examiner scores) was non-significant (adjusted R^2^ = .028, *p* = .071) and step 2 was also non-significant at a trend level (adjusted R^2^ = .062, *p* = .076), with no variable explaining unique and significant BIS-C variance.

**Table 5:** Bivariate and Multiple Regression for Predicting BIS-C Total Score

	Model 1 (*n* = 79)			Model 2 (*n* = 71)		
	Bivariate	Step 1 (Demographic)	Step 2 (+BIS Total)	Bivariate	Step 1 (NIH Examiner scales)	Step 2 (+ RIST)
Socioeconomic Status ^a^	−0.182	−0.110	− 0.070			−0.071
Child Age (years)	−0.201*	−0.338	− 0.454*			−0.508
BIS Total	0.307**		0.363**			
Verbal Fluency Total				−0.231*	−0.095	−0.083
Weighted composite				0.053	0.411	0.306
CPT Total Targets Correct				−0.043	0.007	−0.006
CPT Target Errors				0.270**	0.641	0.785
N-Back Score				−0.238*	−0.537	−0.724
Set Shifting score				−0.070	0.268	0.408
Executive Function Composite Score				−0.214*	0.098	0.208
RISTtotal index IQ ^b^				−0.170		−0.116
Total explained variance (adjusted *R*2)		0.049	0.146		0.028	0.062

Model 1 includes demographic variables (SES and child age) and Parent reported BIS Total

Model 2 includes neurocognitive variables (NIH Examiner scales, RIST)

R-squared values used in this table are adjusted variables

^a^Barratt Simplified Measure of Social Status

^b^Reynolds Intellectual Screening Test Composite IQ is standard score

*p-value < 0.05

**p-value < 0.01

## Discussion

Assessment of child self-reported FI allows for the description of broad domains of functioning from the patients’ point of view, giving valuable information for improvement of health care and resource allocation. We adapted and tested the BIS-C, a multi-dimensional and easy to use self-report measure for children. The current study explored the impact of pediatric cancer upon self-reported FI; and the relationship between self-reported FI and parent-reported child FI and neurocognitive functioning in cancer survivors relative to controls. We found that survivors were similar to controls in self-reported FI. Regardless of group membership, self-reported FI was higher than parent-reported FI, although they were correlated and parent report of FI significantly predicted self-reported FI. Across groups, self-reported FI was associated with four of seven neurocognitive scores.

We hypothesized that based on prior research with FI-related constructs [12–14, 34, 35], BIS-C scores would distinguish pediatric cancer survivors from controls [14]. This was not the case, as we found that the two groups were similar on self-reported FI. Our results align with our previous finding that parent-reported FI did not distinguish between the two groups [33], which may be the product of a variety of methodological issues: low survivor participation rate, lack of treatment intensity heterogeneity, and small sample size. Further, group differences may be more difficult to detect in small samples because such group differences may be based on the relatively small subset of survivors who exhibit impairment, thereby being evident only in large samples [35].

Obtaining child reports of FI provides novel information and identifies a higher rate of FI than does parent report. Thus, across groups (survivor, control), children reported higher levels of FI and higher percentages of clinical caseness (for the higher BIS cutoff (GE 14)) than did their parents, suggesting that children are identifying additional deficits and clinical impairment across domains. The reason children self-report higher levels of FI than their parents’ warrants further investigation. As hypothesized, the two sets of reports were correlated, and parent-report of FI predicted child-reported FI above and beyond child age and SES, but the finding that children report greater levels and greater numbers reaching a clinical cutoff suggest that children are identifying greater FI in themselves than are their parents. In fact, our finding that approximately half of both cancer survivors and controls reported clinically significant levels of FI, in excess of that reported by parents, calls into question the issue of how to address key differences in perspectives between child-reports and parent-proxy reports of function and behavior.

Although we did not find an age effect on BIS-C total, FI decreased with age on two BIS-C subscales: interpersonal relations and self-care/self-fulfillment domain scales. The only significant group differences on BIS-C subscales were between 5–7 years and both 8–10 years and 15–18 years, with 5–7 years reporting greater self-care/self-fulfillment FI. This finding is in the opposite direction of parent-reported FI, wherein we found no significant BIS total difference by age, but the older the child, the greater the *tendency* towards greater BIS (FI), with this difference trending between age 11–14 versus 15–18 years [32]. Examination of parent-reported domain scores between age groups revealed this same pattern, with significant differences between 11 and 14 versus 15–18 age groups in interpersonal relations and self-care/self-fulfillment. Our BIS-C finding may in part be explained by the fact that very young children (5–7 year olds) may have had difficulty with comprehension or answer choice selection, thereby limiting the validity of their self-report. While both child and parent reports of total FI did not distinguish survivors from control children, they suggest modest but opposite age-related patterns of impairment, particularly in interpersonal relations and self-care/self-fulfillment domains. This age-related issue and questions about instrument validity with younger children warrant additional investigation.

We found that neurocognitive functioning was modestly related to FI, with four of seven Examiner scales correlating with FI in the predicted directions. This finding aligns with our previous parent-reported FI finding that several neurocognitive measures were related to both total and subscale BIS scores for both cancer survivors and controls [33]. There clearly is a link between parent-report of psychosocial adaptation and survivor EF, particularly among children with Central Nervous System (CNS) tumors [36]. Survivors of CNS tumors have demonstrated that cognitive and emotional functioning are predictors of quality of life [37]. Further, in a study of older adults, functional capacity correlated positively with cognitive variables, and each type of functional capacity was predicted by somewhat different cognitive variables [38]. Our significant correlations became non-significant when age was controlled, suggesting that age explains a great deal of the shared variance of the BIS-C and Examiner scores.

Moreover, our findings of age-related differences suggest that child and parent perspectives change as children grow older. This critical distinction in perspectives on function and how they are divergent is important in the subsequent design of intervention studies for helping children improve their day-to-day function. Psychosocial interventions that solely focus upon parent perception are likely to miss children who may be in distress secondary to their perception of increasing functional difficulties. Perhaps a solution to this dilemma is to capitalize upon both parent and child perceptions of day-to-day functioning by using a discrepancy between these perspectives for a specific psychosocial intervention and, conversely, using a similarity in perception for a completely different intervention. Although this study provides evidence of the importance of child perspectives to pediatric outcomes research, exporting such a measure to another culture may bring challenges if a child’s voice in health care is perceived or valued differently.

### Study limitations

A relatively small and heterogeneous sample of cancer survivors with a low participation rate, limited treatment intensity with an average “medium” treatment intensity, a large range in time since treatment, and a cross-sectional design limit generalizability and inferences of causality. In large part, as a function of our small cancer survivor sample size characterized by relatively low treatment intensity, we found no FI difference between cancer survivors and controls. It is possible that FI is not a distinguishing characteristic for our sample, with other researchers observing mixed results when evaluating FI as an outcome [4, 39, 40].

The low recruitment rate of cancer survivors (24%) speaks to the possibility that survivors who agreed to participate differed systematically from those who were approached but declined participation. To the extent that relevant variables were available for non-participants, a selection bias was evaluated. Non-participant data were limited to child age, sex, and diagnosis. Based on these variables, females were more likely to participate than males, indicating a relative overrepresentation of female cancer survivors. There were no significant differences in age and diagnosis between participants and non-participants. However, there were additional variables that may have affected study participation that were not evaluated. Finally, our BIS-C age-related findings raise questions about instrument validity with younger children, warranting additional investigation.

## Recommendations for research and practice

Obtaining reports of functional impairment from youth and parents is an emerging research area [40]. Our limited sample of pediatric cancer survivors indicates a need for improvement in this line of research, including larger and more diverse samples, along with psychometric analyses that provide greater clarity to the response characteristics of a functional impairment measure. Certainly there is a need for a validated tool for screening and monitoring the health of pediatric oncology survivors, evident in the guidelines from the Children’s Oncology Group [41]. Our research and that of others is pointing to the need to ascertain child functional impairment from both parent and child perspectives. A tool that can assess functional impairment has the potential for many uses, including population and clinical screening, acute impact of natural disaster assessment, as well as intervention monitoring. With further development, functional impairment appears to have the potential to provide a robust target for clinical intervention.

## Conclusions

In conclusion, the BIS-C appears to be an adequate screening tool to identify self-reported FI among children ages 5–18, with some validity concerns in children ages 5–7. These preliminary results of this measure may add to the picture currently obtained by parent-reported FI, in that although there is substantial agreement and overlap between self-reported and parent-reported FI, there is also unique information provided by the BIS-C. Specifically, our finding that children report greater total and subscale FI, as well as greater numbers reaching a clinical cutoff (when set high) compared with their parents’ reports, warrants further investigation to determine what impairments children are identifying that parents are not. Additional validation studies of the BIS-C are warranted, particularly among younger children. In conclusion, pediatric cancer survivors should be screened for psychosocial functioning based on risk- and exposure-related guidelines [41].

## Data Availability

Data and material are available for review and replication purposes.

## Abbreviations

ALL: Acute Lymphoblastic Leukemia
BIS: Brief Impairment Scale
BIS-C: Brief Impairment Scale-Child Version
CCSS: Childhood Cancer Survivor Study
CPT: Continuous Performance Task
CNS: Central Nervous System
FI: Functional Impairment
RIST: Reynolds Intellectual Screening Test
SES: Socioeconomic Status
